# A Double-Edged Sword: Aneuploidy is a Prevalent Strategy in Fungal Adaptation

**DOI:** 10.3390/genes10100787

**Published:** 2019-10-10

**Authors:** Hung-Ji Tsai, Anjali Nelliat

**Affiliations:** 1 Institute of Microbiology and Infection, and School of Biosciences, University of Birmingham, Edgbaston, Birmingham B15 2TT, UK; 2 Department of Pathology, School of Medicine, Johns Hopkins University, Baltimore, MD 21205, USA; 3 Department of Systems Biology, Harvard Medical School, Boston, MA 02115, USA

**Keywords:** aneuploidy, stress adaptation, genome plasticity, chromosome instability

## Abstract

Aneuploidy, a deviation from a balanced genome by either gain or loss of chromosomes, is generally associated with impaired fitness and developmental defects in eukaryotic organisms. While the general physiological impact of aneuploidy remains largely elusive, many phenotypes associated with aneuploidy link to a common theme of stress adaptation. Here, we review previously identified mechanisms and observations related to aneuploidy, focusing on the highly diverse eukaryotes, fungi. Fungi, which have conquered virtually all environments, including several hostile ecological niches, exhibit widespread aneuploidy and employ it as an adaptive strategy under severe stress. Gambling with the balance between genome plasticity and stability has its cost and in fact, most aneuploidies have fitness defects. How can this fitness defect be reconciled with the prevalence of aneuploidy in fungi? It is likely that the fitness cost of the extra chromosomes is outweighed by the advantage they confer under life-threatening stresses. In fact, once the selective pressures are withdrawn, aneuploidy is often lost and replaced by less drastic mutations that possibly incur a lower fitness cost. We discuss representative examples across hostile environments, including medically and industrially relevant cases, to highlight potential adaptive mechanisms in aneuploid yeast.

## 1. Introduction

Aneuploidy, an unbalanced genomic state with gain or loss of chromosomes, has been observed across eukaryotic organisms and often leads to severe fitness cost and developmental defects [1,2,3,4,5]. While an aneuploid genome encompasses thousands or millions of possible numerical combinations of chromosomes during cell divisions, each aneuploid genome type may confer a unique phenotypic profile [6], and thus, it is nearly impossible to approach the “genotype-to-phenotype” landscape comprehensively. For instance, budding yeast *Saccharomyces cerevisiae* has 16 chromosomes in a haploid genome; there can be, in theory, 65534 possible aneuploid karyotypes (2^16^-2) when the chromosome copy number variation is limited to only one or two copies. The numerous chromosome combinations and the ongoing genome instability limit our ability to define the phenotypic characteristics of aneuploidy. The earliest studies of the phenotypic consequences of aneuploidy were in the flowering plant, *Datura*, where aneuploids, particularly those with lower basal ploidy levels, were severely defective [7,8]. Many human diseases, such as Down syndrome and cancer, are attributed to aneuploidy [9,10,11,12,13], and paradoxically, the aneuploid state frequently exists in normal neuronal cells and hepatocytes in mammals [14,15,16,17]. In eukaryotic microbes, aneuploidy enables rapid adaptation to environmental challenges by conferring genomic and phenotypic variation [6,18,19,20,21,22,23,24]. In this review article, we will highlight the current perspective and the examples of aneuploidy providing fungal species survival niches under various environmental conditions.

## 2. Cellular Impacts of Genome Aneuploidization

**Figure 1:**
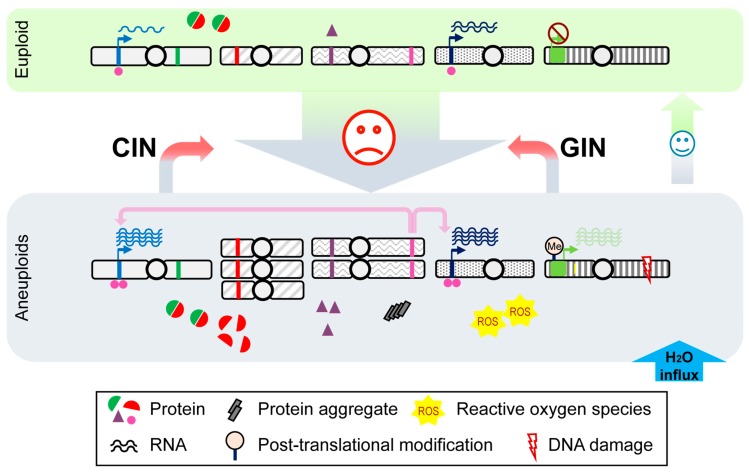
A brief overview of cellular impacts from genome aneuploidization. Aneuploidization may occur when cells are under unfavorable conditions, leading to large-scale gene copy number variation. An overproduction of proteins from genes on aneuploid chromosomes causes proteome imbalance, while an excess flux of free protein subunits (e.g., red semicircles), which are not assembled into protein complexes with well-defined stoichiometry (red + green semicircles), could perturb the proteostasis network. The proteasome system could be overwhelmed due to the needs of degrading excess proteins (e.g., red semicircles and purple triangles). Increased loads of protein aggregates (grey rectangles) can be detected as a consequence of reduced Hsp90 folding capacity in aneuploidy. ROS levels are also increased, leading to oxidative stress. In addition, these extra protein subunits increase intracellular osmolarity triggering an influx of water (blue arrow), that leads to hypo-osmotic stress. As a result, an increased turgor pressure causes membrane stress. At the level of transcription, genes from euploid chromosomes (blue and dark blue bands), targeted by transcription factors encoded on aneuploid chromosomes (pink bands), could also be overexpressed; in parallel, aneuploidy may change epigenetic landscape, such as post-translational modifications (light orange flag on a gene labeled with green band), in a genome-wide manner to affect gene expression (green bands). As aneuploidy is a direct outcome of genomic instability (GIN) or chromosomal instability (CIN), it could drive further GIN and CIN from defective DNA damage repair and mitotic errors and may exacerbate the existing defective phenotypes. On the other hand, mitotic stress from GIN and CIN may continuously generate karyotype diversity in the cell population for adaptive phenotypes under stressful conditions. Once the stress is withdrawn or a gene mutation with relatively minor fitness trade-off occurs, cells may revert to the euploid state.

In the past two decades, extensive investigations in *S. cerevisiae* have revealed several stress signatures in aneuploid cells [1,6,25,26,27,28,29,30,31]. A panel of disomic yeast strains were first analyzed by Amon and colleagues, systematically showing proliferative defects resulting from cell cycle delays, persistent DNA damage, elevated levels of reactive oxygen species and hyper-susceptibility to particular reagents, such as cycloheximide and MG132 interfering with translation and the proteasome, in several disomic aneuploidies (Figure 1) [1,26,28,29,32,33]. These phenotypes result from an overproduction of proteins coded on aneuploid chromosomes, especially those proteins in multi-subunit complexes with well-defined stoichiometry [34]. Indeed, gain of a specific chromosome alone, encoding dosage-sensitive genes, causes lethality in yeast due to stoichiometric imbalance (e.g., copy number gain of *TUB2* on aneuploid chromosome VI) [35]. Further, many of these aneuploid strains exhibiting proteotoxic stress are prone to Hsp104-associated protein aggregations while excess proteins overload the protein quality control systems and titrate protein chaperons, causing the accumulations of damaged or unfolded peptides [28]. A loss of function mutation in *UBP6*, encoding a deubiquitinating enzyme, improves the proliferative rates of four tested disomic strains (gain of chromosome V, VIII, IX and XI). The impacts of altered proteome in these disomic strains were attenuated in the absence of *UBP6* function either by enhanced degradation of excess proteins or by increased clearance of protein aggregates [1,25,28]. Additionally, another deubiquitinase enzyme *UBP3*, involved in vesicle transport, was identified in a genome-wide screen as a gene which upon deletion impairs the fitness of several disomic strains (gain of chromosome II, XI, XII, XIV, XV and XVI) [30]. Taken together, proteotoxic stress is observed in many analyzed disomic strains (Figure 1), but not in all disomic strains or yeast with more complex aneuploid stoichiometries. More transcriptomic analyses were performed in selected aneuploid strains, and most disomic yeast (W303 laboratory strain background) exhibited a transcriptional profile similar to the environmental stress response (ESR), a general expression signature triggered by diverse stresses in yeast [36]. However, this transcriptional profile was not observed in all disomic yeast (F45 strain background) [37], wild yeasts [38] or yeast populations generated by random aneuploidization [31].

In addition to the well-recognized proteotoxic stress in selected aneuploid strains from considerable studies, a common physiological change associated with aneuploid cells harboring diverse aneuploid karyotypes has been identified in a recent study from Li and colleagues [31]. Transcriptomic analyses were performed from mixed aneuploid populations, including thousands of meiotic progeny of triploid yeast, with random karyotypes, where the karyotype-specific expression is minimized. By comparing the genomic expression pattern in aneuploid populations with those of cells under diverse stress conditions, the aneuploidy-associated transcriptome resembling hypo-osmotic stress responses was uncovered as a common biophysical signature in aneuploid cells irrespective of their aneuploid karyotypes. This osmotic imbalance is a direct impact of the unbalanced proteome caused by excessively produced proteins, especially unassembled protein subunits from complexes, that increase intracellular osmolarity (Figure 1). Consequently, an increased intracellular turgor due to water influx in aneuploid cells results in cell surface stress, opposing endocytic machineries and further desensitizes the turnover of plasma membrane transporters in response to metabolic cues. The aneuploid phenotypes under this hypo-osmotic stress state also renders a genetic dependence on the ART-Rsp5 mediated endocytic pathway to modulate intracellular homeostasis [31]. The study, for the first time, reveals a common signature in aneuploid cells with random karyotypes. Yet, it remains elusive how the aneuploid state with its mostly negative effects, contributes to the high cell adaptability to harsh environments.

## 3. Aneuploid Fungi Are Widespread in Diverse Environments

Ploidy variation is widely observed in fungal species across wild, clinical and laboratory environments [39]. Petrov and colleagues showed that, of 145 diploid *S. cerevisiae* MA lines (mutation accumulation) evolving for an average of 2062 generations each, 31 sequenced strains were aneuploid (29 gain and two loss of chromosome events), suggesting aneuploidy likely occurs frequently in an evolutionary time scale [40]. Wild yeast isolates from Evolution Canyon, Israel, displayed tolerance to multiple environmental stresses, including high multi-metal abundances, UV radiation and oxidative stress, and further phenotypic analyses of selected adaptive phenotypes are associated with aneuploidy and other large-scale chromosomal rearrangements [41,42,43]. For instance, natural isolates showing high tolerance to copper and cadmium carried segmental aneuploid chromosomes, and the rearranged chromosomes frequently reverted back to euploid when the stress was relaxed [43]. This phenotype of losing segmental aneuploid chromosomes under a less selective environment suggests that aneuploidy drives adaptation but meanwhile leads to fitness cost. Gasch and colleagues surveyed 47 non-laboratory yeast including 15 wild isolates (plant, soil and insects), 19 industrial isolates (fermentation, beer, wine and bakery), 12 clinical isolates and one undocumented isolate [38]. Nearly a third of sequenced strains were aneuploid (three in wild isolates, eight in industrial isolates and three in clinical isolates), and these natural aneuploidies showed minimal fitness cost, in contrast to laboratory aneuploid strains [1,38]. As such, aneuploidy is well tolerated in wild *S. cerevisiae* and can be leveraged to rapidly adapt to unexpected stresses in the wild. Yet, the cellular mechanisms of how these stable aneuploid genomes cope with natural environmental stress remain unclear, since the investigations in the laboratory cannot fully recapitulate their evolutionary history. 

Clinical isolates of human pathogenic fungi, such as *Candida* and *Cryptococcus* species, have been intensively studied in light of the tight relationship between large-scale genome instability and antifungal resistance [44]. Copy number variations associated with phenotypes of antifungal resistance were first detected in clinical *Candida glabrata* isolates, harboring aneuploid chromosomes [45,46,47]. A direct link between aneuploidy and adaptive drug resistance was confirmed in another pathogenic fungus, *Candida albicans*, isolated from bone marrow transplant (BMT) patients [18]. In this panel of clinical isolates, aneuploid isochromosome 5 (i5L) was frequently observed from aCGH microarray analyses, and additional experiments showed that gene amplifications of *ERG11* and *TAC1* on the i5L chromosome drives resistance to the commonly used azole drug, fluconazole [48]. Further investigations tracking the evolution of clinical fungal isolates in chronological order found that drug resistance-associated mutations are likely fixed in the adapted population while aneuploidy was mostly transient [49]. This suggests that aneuploidy provides a unique niche during the evolution of drug resistance, mostly a rapid solution for coping with the antifungal stress in vivo. Experimental evolution, in parallel, supports these observations by acquiring the same aneuploid chromosomes rendering fluconazole resistance, and of note, i5L could appear in less than four generations, indicating aneuploidization drives rapid adaptation to antifungal stress [50]. Moreover, karyotypic analyses in clinical *Candida auris*, a known multidrug resistant fungus, also showed ongoing, massive chromosomal rearrangements or possibly a transient aneuploid state, with near-haploid genome content, supporting the idea that aneuploidy could be an intermediate status promoting adaptation [51]. *Cryptococcus* species also leverage aneuploidy or other large-scale genome instability events to obtain growth advantage under antifungal stress. *C. neoformans*, by gaining additional chromosome 1 or 4, confers fluconazole resistance phenotypes [23,52,53]. Meanwhile, polyploid *C. neoformans* titan cells, resistant to host stress, can produce aneuploid progeny when exposed to fluconazole, but generate haploid progeny under an optimal growth condition, indicating a selective preference of aneuploid state for antifungal adaptation [54]. *S. cerevisiae*, a widely used fungus in commercial products, may evolve under host stress and become pathogenic, with increased virulence, to the host. More than a third of clinical *S. cerevisiae* strains were identified as aneuploid (36% of 144 sequenced yeast strains, 132 from clinical isolates), while the other third was polyploid (3N or 4N). The high ploidy variations in these isolates could be explained by the frequently observed *NDC80* mutation, an essential gene for chromosomal fidelity [55]. Therefore, aneuploidization (or polyploidization) events in *S. cerevisiae* may drive phenotypic switch, from commensal to pathogenic in the host environment. In contrast, specific aneuploid karyotype may also promote commensalism balanced by a myriad of host stress including immune responses [56].

Experimental evolution has enabled the exploration of evolutionary trajectories with controllable environmental parameters, such as limited or undesired nutrition resources, antifungal compounds, extreme temperatures and those mimicking wild or host environments, to track how cells adapt to stress on an evolutionary time-scale. Evidently, aneuploid yeast emerge rapidly from different adverse environments, and specific aneuploid karyotypes drive adaptive phenotypes in the context of certain stress conditions (Table 1) [6,24,57,58,59,60,61,62,63,64,65,66,67,68,69,70,71,72]. For example, laboratory haploid and diploid *S. cerevisiae* (S288c background) growing in glucose-limited and phosphate-limited chemostats for over 200 generations, substantial aneuploidy was detected in many adaptive diploids, exhibiting selective advantages, consistent with earlier observations in a glucose-limited condition [59,73]. In another example, tetraploid, diploid and haploid cells were passaged in a poor carbon source, raffinose, and aneuploid chromosome XIII was observed to confer a significant fitness increase to tetraploid strains in raffinose, but not in glucose, while diploid and haploid cells did not acquire aneuploidy during the experiments, echoing the specific, context-dependent nature of aneuploidy [66]. Of note, when stress was applied gradually rather than abruptly, aneuploidy did not emerge [62], reinforcing the idea that aneuploidy is more likely a high-stakes adaptive tactic rather than a fine-tuned strategy. Taken together, aneuploidy enables broad and rapid traversing of the search space for adaptive phenotypes by introducing drastic copy number changes in a significant fraction of the genome [74].

## 4. Gene Copy Number Variations on Aneuploid Chromosomes Drive Adaptive Phenotypes

A classic example of aneuploidy driving an adaptive phenotype, such as antifungal resistance, with a detailed mechanistic explanation, is in *C. albicans*, characterized by Berman and colleagues (Table 1) [18,48,50,85,86]. Clinical isolates acquire resistance to fluconazole by gaining aneuploid chromosomes with extra copies of *ERG11* encoding lanosterol-14-α-demethylase targeted by fluconazole, and *TAC1* encoding a transcriptional activator of drug efflux pumps *CDR1* and *CDR2* on euploid chromosome 3. *ERG11* copy number is positively correlated with the levels of fluconazole resistance, while increased *ERG11* mRNA expression was observed in 35% of fluconazole resistant *C. albicans* [48,87]. In parallel, a hyperactive *TAC1* allele driving high levels of *CDR1* and *CDR2* expression, was also found on isochromosome 5L with high copy numbers [48,85]. These two distinct pathways can lead to fluconazole resistance on a similar level, and in these clinical isolates with gain of chromosome 5L, they additively, yet independently, affect the adaptive phenotypes of fluconazole resistance [48]. Meanwhile, the upregulated genes and pathways (targets of *TAC1*, including *CDR1/2*) are on multiple chromosomes across the genome [88], indicating that phenotypic changes in aneuploidy could be due to either direct or *trans*-acting effects from drastic gene copy variations on aneuploid chromosomes. Moreover, gene copy variations from aneuploid chromosomes could allow a broad “phenotypic space” during adaptive process and thereby afford the potential for the selected karyotype in one environment to dictate adaptation in another environment. For instance, a single karyotype, trisomic chromosome 2, can confer cross adaptation to multiple unrelated drugs, as in the case between the treatments of anticancer drug hydroxyurea and antifungal drug caspofungin, in *C. albicans* [76]. Copy number gains of *RNR1* and *RNR21* genes conferred the adaptation to hydroxyurea and meanwhile, independent of the hydroxyurea-resistance pathways, increased survival on caspofungin was observed in the presence of the same chromosome 2 gain by a Crz1-independent pathway. However, the gene specific mechanism of trisomic chromosome 2 for adapting to caspofungin remains unknown.

Further investigations to understand how antifungal stress leads to aneuploidization in *C. albicans* reveal that the presence of fluconazole impedes cell cycle progression and causes cytokinesis failure [89]. In a subpopulation of yeast exposed to fluconazole for 12 hours, DNA content increased with abnormal cell morphology during cell division, and these cells later become intermediate polyploid “trimeras”. Following subsequent unequal segregation with multiple spindles for 1-3 cell divisions, trimeras could yield viable aneuploid progeny with complex karyotypes that remain unstable with ongoing chromosome instability. The “trimera” state can be found from cells under various types of azole exposures and was also observed in non-*albicans* species, such as *C. glabrata* and other CUG-clade *Candida* species [89]. Complex aneuploid karyotypes can also be observed in *C. glabrata*, which carries chimeric chromosomes associated with high tolerance of fluconazole concentration [47,90]. Gain of small or chimeric chromosomes increases copy numbers of genes such as *PDH1* (a paralog of *CDR1* multidrug ABC transporter and an ortholog of *PDR5* in *S. cerevisiae*), that are associated with antifungal resistances. However, this mechanism of aneuploidization via perturbed cytokinesis is not universal to fungal species, such as more distantly related fungi, *S. cerevisiae* [89] and *C. neoformans* [91]. A subsequent study showed that during fluconazole treatment, uninucleate haploid *C. neoformans* produced aneuploid progeny through asymmetric budding processes as a primary mechanism of aneuploid formation, while multinucleated cells were only detected in a low frequency [91,92]. The levels of fluconazole resistance were correlated with the gene dosage effects of *ERG11* and *AFR1*, encoding an ABC transporter on chromosome 1. While rare intermediate polyploid “trimera” was observed, polyploid *C. neoformans* “titan cells”, can also acquire aneuploidy to gain advantageous growth under fluconazole treatment [54]. Nevertheless, the possible mechanisms, such as the events of endoduplication or mitotic error, yielding asymmetric aneuploid progeny resistant to azoles in *C. neoformans* and *S. cerevisiae* remain largely undetermined. In addition, aneuploidy is observed as an intermediate state when ploidy reduction happens during the parasexual cycle in *C. albicans* [93,94,95,96]. Tetraploid and aneuploid cells could return to a diploid or near diploid state via concerted chromosome loss, concomitant with recombination events, such as loss of heterozygosity, that could reveal a number of recessive traits. As such, both aneuploid intermediates and their subsequent diploid progeny could facilitate adaptive phenotypes through diverse genetic variations produced by parasex. 

Another mechanism leading to copy number variations and aneuploidy is through massive genomic rearrangements through repeat sequences, characterized by Selmecki and colleagues [97]. By identifying and analyzing long repeat sequences across the *C. albicans* genome in 33 clinical and experimentally evolved isolates, long-range genomic rearrangements through these long repeat sequences can form copy number variations, segmental aneuploidy and loss of heterozygosity as a source of genome plasticity. Consistent with the above example of i5L formation, long inverted repeat sequences within *CEN4* (and *CEN5* in i5L formation) can result in isochromosome 4R leading to increased fluconazole resistance [97]. In contrast, laboratory *S. cerevisiae* often uses short inverted repeat sequences generating copy number variations, including amplifications of segmental chromosomes, to adapt to stresses [72,98]. 

There is ample evidence of the selective advantage conferred by the gene dosage-based mechanism of aneuploidy from experimental evolution in the laboratory and analyses of natural isolates (Table 1). Under copper-rich conditions, the majority of both evolving *S. cerevisiae* exhibit aneuploidy of chromosomes II, VII or VIII, that provide extra copies of *SCO1/2* which functions in transport of copper to cytochrome c oxidase in the mitochondrial inner membrane, *CUP1*, which encodes a metallothionein and *CUP2*, which activates transcription of *CUP1*, respectively [41,43,65]. Studies employing nutrient-deprivation selections revealed similar principles—specific chromosomes harboring genes that facilitate the uptake or metabolism of the limiting factor are upregulated often through gene amplification [59,73]. Additionally, specific aneuploid karyotypes confer adaptation to high temperature, pH and ER stresses by virtue of extra copies of few specific genes on the aneuploid chromosomes [62,71]. For instance, Beaupere et al. [71] demonstrated that resistance to tunicamycin-induced ER stress attributed to the gain of an extra copy chromosome II, could be narrowed down to the combined effect of three genes involved in regulation of protein folding and degradation, *ALG7*, *PRE7* and *YBR085C-A*, that are a GlcNAc-1-P transferase involved in synthesis of oligosaccharide precursor for N-linked protein glycosylation, a subunit of the 20S proteasome and an uncharacterized protein speculated to be a UPR signaling factor, respectively.

In the few cases where the mechanism by which aneuploidy affects relevant traits of industrial yeast strains were uncovered, gene dosage effects were prominent (Table 1). In wine yeast, an increased copy number of chromosome VII, which carries the alcohol dehydrogenase genes *ADH2* and *ADH3* is correlated with increased capacity to oxidize ethanol during fermentation [82]. In lager brewing strains of *S. pastorianus*, production of diacetyl, an unwanted byproduct that is removed at the end of fermentation, was correlated with aneuploidy of chromosomes III, VIII, X, XII, and XIV [80]. These chromosomes harbor genes involved in valine biosynthesis, which generates the precursor for diacetyl production. Similarly, in a triploid industrial *S. cerevisiae* strain, isolated from corn mash used in a Chinese bioethanol factory, a clone possessing an extra copy of chromosome XI displayed improved ethanol yield. Further experiments indicated that the altered expression activity of some genes involved in ergosterol synthesis on the aneuploid chromosomes partly explained the improvement [83].

While there is limited understanding of the role of aneuploidy in plant pathogenic fungi, some of these phytopathogens are known to possess multiple dispensable chromosomes called accessory chromosomes that are known to harbor virulence loci [99]. In one such fungal species, *Fusarium oxysporum* f.sp. *lycopersici (Fol)*, a tomato pathogen, supernumerary chromosome 14 can be transferred between strains, converting a non-pathogenic strain into a pathogen. Interestingly, genes for key proteins secreted during the spread of *Fol* in the tomato xylem involved in virulence (Six1, Six3) and the putative oxidoreductase gene, *ORX1* are encoded on chromosome 14 [78]. Another pathogen *Nectria haematococca*, possesses an accessory chromosome harboring the gene *Pda6*, a pisatin demethylase that allows detoxification of pisatin, an antimicrobial compound produced by garden pea plants [79]. Recently, sequencing of the single-spore isolates of a vegetable pathogen *Phytophthora capsica* revealed heterogeneous, drastic, and stable aneuploidy. This dynamic, extreme aneuploidy was speculated to confer observed resistance to mefenoxam, a commonly used phytopathogenic toxin [100]. In contrast, in isolates of the sudden oak death pathogen *Phytophthora ramorum*, aneuploid isolates exhibited slower growth and lower pathogenicity compared to wild-type isolates, reiterating the dichotomous nature of aneuploidy in host-pathogen interactions [101].

Apart from changes at the single cell level, aneuploidy can confer changes in complex, multicellular phenotypes like colony morphology. Aneuploidy in wild yeast strains is often associated with morphological changes, including elongated pseudohyphal growth and filamentous colonies. Dudley and colleagues demonstrated that the gain of a single chromosome in a strain of *S. cerevisiae* was sufficient to switch colony morphology from the “fluffy” to the “smooth” state [21,37], and its subsequent loss, to revert the strain back to the fluffy state. Reducing the copy number of *DIG1*, a transcription factor present on the aneuploid chromosome and involved in the regulation of certain mating type specific genes partially converted the smooth morphology of the disomic cells back to fluffy, while mild overexpression of *DIG1* in the euploid, reverted the morphology in the opposite direction. However, the partial phenotypic switching indicates that other genes on the aneuploid chromosome might also be involved. Other community phenotypes such as flocculation and sedimentation are essential steps of cell aggregation during the brewing process and were positively correlated with copy numbers of chromosomes which harbor flocculin genes *LgFLO*, *FLO1*, *FLO5* and *FLO10* in industrial yeast strains [80].

In addition to the direct effects of altered gene copy numbers in a context dependent manner, the phenotypic switch in aneuploid cells may also result from epigenetic changes. A disomic yeast with gain of chromosome X as well as other complex aneuploid karyotypes can impair epigenetically-controlled transcriptional silencing at the mating-type loci [102]. This silencing defect could be due to the nuclear positioning of the silent chromatin and may involve at least four genes working synergistically, but not necessary functionally related, indicating a regulatory mechanism, indirect or beyond copy number variations, caused by aneuploidy. Lastly, since aneuploidization creates possibly unlimited phenotypic traits for adaptation to life-threatening stresses, it is likely that cells may evolve novel phenotypes that are subsequently selected to cope with stress, rather than using existing stress response pathways. For instance, Rancati et al. demonstrated that in the absence of an essential gene *MYO1* which is required for cytokinesis, rare *∆myo1* survivors with restored cell division emerged via acquiring specific aneuploid stoichiometries [60]. This violation of supposed ‘essentiality’ was not recovered by restoring the original cytokinesis machinery when *MYO1* was reintroduced, but instead involved genes from seemingly unassociated pathways—a transcription factor, *RLM1* and a kinase, *MKK1*, both involved in maintaining cell wall integrity. This suggests that apart from gene dosage based adaptive phenotypes, aneuploidy could potentially be a springboard for neofunctionalization. However, how cells remodel the cell wall to cope with the changes in cytokinesis during aneuploidization remains unclear. Furthermore, Rancati and colleagues found that 9% of the conventionally defined “essential” genes could be overcome through adaptive evolution and ploidy variations, including aneuploidy, reinforcing that it is a prevalent adaptive strategy [103].

## 5. Future Perspective

The balance between genome plasticity and stability is fine tuned to ensure cells survive and propagate in a fluctuating or an unpredictable environment, such as in the wild or a host. Cells often leverage aneuploidization, as a rapid and reversible strategy, to cope with diverse stress conditions. In this review, we have provided representative examples and potential mechanisms to highlight how aneuploidy contributes to fungal cell adaptation under various stress conditions. In addition to gene copy variations, aneuploidy itself, even within the same karyotype, may display non-genetic heterogeneity in response to environmental perturbations [104]. Thus, when mitotic error occurs, random aneuploid karyotypes could provide the cell population numerous phenotypic traits, including detrimental or beneficial individuals, to ensure the survival of progeny even in the most taxing environments. Remarkably, this “gambling” strategy is not leveraged when the stress is applied gradually rather than abruptly, and cells often revert to euploid once the stress is relieved or when a beneficial mutation emerges, as evident in both laboratory yeast and clinical isolates [43,49,62]. The detailed mechanisms of adaptive evolution driven by aneuploidy are elusive, and it remains to be seen whether there is a molecular mechanism allowing cells to sense aneuploidization. How do aneuploid cells under stress mitigate negative effects from hundreds of gene copy number variations if only few contribute to the adaptive phenotypes? At the molecular level, questions on how stress conditions lead to chromosome instability (CIN) also remain largely unanswered, although CIN genes have been intensively investigated by many research groups [105,106,107,108,109,110,111].

Importantly, antifungal resistance is a rapidly emerging problem, and one of the contributing mechanisms is genome instability, including many chromosomal-scale events. Strategies to effectively target aneuploid fungi are becoming an obvious need in medicine. As such, genetic screens were performed to identify a “magic bullet” targeting aneuploidy in different laboratory conditions [31,112,113]. While the impact of aneuploidization is genome-wide, and it affords a high degree of genetic variation, it is difficult to eliminate aneuploid fungi and to avoid antifungal adaptations. However, a proof-of-principle screen demonstrated that cells are steered towards specific aneuploid karyotype under certain antifungal stress, and a subsequent chemical screen that was performed eliminated the homogenized aneuploid population, known as a strategy of “evolutionary trap” [114]. In parallel, a biophysical signature, hypo-osmotic stress state, was reported recently as a phenotypic commonality in aneuploid yeast populations with random karyotypes, that could be the Achilles’ heel of aneuploidy [31]. The work, along with other efforts in the field may provide clinically useful insights in the foreseeable future. Yet, these studies also raise more questions of cellular evolution during the adaptive process, particularly with regard to the ultimate goal of blocking aneuploidy-driven antifungal adaptation. For instance, is there an absolute essentiality of cellular functions in aneuploid cells given that we never observed a lethal phenotype through gene deletion screens in aneuploid cells? Taken together, it is clear that while aneuploidy is a widespread and common phenomenon across different fungal species and other eukaryotes, more in-depth investigations are required to understand its fundamental biology and bring us closer towards solving the clinical problems in aneuploid diseases.

## Figures and Tables

**Table 1:** Examples of aneuploidy acquired under diverse stresses across different fungal species.

Species	Strain Details	Selected Phenotype	Aneuploid Chromosomes	Implicated Genes	Aneuploidy Transience	Reference
*C. albicans*	Clinical isolates	Fluconazole resistance	V (+1) *	*ERG11, TAC1*		Selmecki et al. (2008) [48]
	In vivo OPC mouse model	Host defense during OPC	VI (+1)			Forche et al. (2019) [56]
	Laboratory strains	Cisplatin resistance	V (−1), II (+1), II (+2)			Yang et al. (2013) [75], Yang et al. (2019) [76]
		Hydroxyurea resistance	II (+1)			Yang et al. (2019) [76]
		Suppressor of *RGD1* deletion	III (+1) *, VII (+1)	*NPR2*		Mount et al. (2018) [77]
	Multiple clinical isolates	Fluconazole resistance	V (+1) *, III (+1) *	*ERG11, MRR1, CDR1, CDR2*	Confirmed	Ford et al. (2015) [49]
*C. neoformans*	Clinical isolates	Fluconazole heteroresistance	I (+1), IV (+1)	*ERG11, AFR1*		Sionov et al. (2010) [23], Sionov et al. (2013) [53]
	Laboratory strains	Fluconazole heteroresistance	I (+1), IV (+1)	*ERG11, AFR1, SEY1, GLO3*		Ngamskulrungroj et al. (2012) [52]
*F. oxysporum*	Plant pathogen	Increased pathogenecity	XIV (+1) **	*SIX1, SIX3, ORX1*		Ma et al. (2010) [78]
*N. haematococca*	Plant pathogen	Increased pathogenicity	CD (+1) **	*PDA6*		Miao et al. (1991) [79]
*S. pastorianus*	Industrial lager strains	Flocculation	I (+1), IV (+1), X (+1), XII (+2)	*LgFLO, FLO1, FLO5, FLO10*		Van den Borek et al. (2015) [80]
		Increased diacetyl synthesis	X (+1), XII (+2)	*ILV5, ILV3*		Van den Borek et al. (2015) [80]
*S. cerevisiae*	Clinical isolates	Host survival	variable across strain phylogeny			Zhu et al. (2016) [55]
	Environmental isolates	Copper tolerance	II (+1), VII (+1), VIII (+1)	*CUP1, CUP2, SCO1, SCO2*		Ezov et al. (2006) [41], Chang et al. (2013) [43]
		Freeze-thaw tolerance	XII (+1)	*AQY2*		Hose et al. (2015) [38]
	Industrial isolates	Ethanol tolerance	III (+1)			Morard et al. (2019) [81]
	Industrial wine strain	Increased ethanol oxidation	VII (+1), XIII (+2)	*ADH2, ADH3*		Guijo et al. (1997) [82]
	Industrial beer strain	Increased ethanol yield	XI (+1)			Zhang et al. (2015) [83]
	Laboratory strains	4-NQO resistance	IV (+1)	*ATR1*		Pavelka et al. (2010) [6]
		Benomyl resistance	XII (−1)			Chen et al. (2012) [61]
		Copper tolerance	II (+1), VIII (+1)	*CUP1, SCO1, SCO2*		Gerstein et al. (2015) [65]
		Ethanol tolerance	III (+1), VIII (+1)			Voordeckers et al. (2015) [68]
		Ferulic acid tolerance	XIV (+1)			Sato et al. (2014) [63]
		Flocculation	I (+1)	*FLO1*		Hope et al. (2017) [70]
		Fluconazole resistance	VIII (+1)	*ERG11*		Chen et al. (2012) [61]
		Galactose tolerance	VIII (+1)	*GAL80*		Sirr et al. (2015) [67]
		Glucose-limited growth	I (+1), III (+1), V (+1) *, XIV (−1)		Speculated	Gresham et al. (2008) [59]
		Nitrogen-limited growth (glutamine)	XI, XI (+1–+4) *	*GAP1*		Lauer et al. (2018) [62]
		Heat shock tolerance	III (+1)	*17 genes*	Confirmed	Yona et al. (2012) [62]
		High pH tolerance	V (+1)		Confirmed	Yona et al. (2012) [62]
		Phosphate-limited growth	IV (+1), VI (+1), X (+1), XIII (+2), XVI (+1)		Speculated	Gresham et al. (2008) [59]
		Raffinose growth	XIII (+1)			Selmecki et al. (2015) [66]
		Radicicol resistance	XV (+1)	*STL1, PDR5*		Chen et al. (2012) [61]
		Suppressors of *MEC1* deficiency	IV (+1)	*RNR1*		Gasch et al. (2001) [58]
		Suppressors of *MYO1* deletion	XIII (+1), XVI (+1)	*RLM1, MKK2*		Rancati et al. (2008) [60]
		Suppressors of *RPS24A* and *RNR1* deletion	IX (+1)	*RPS24B, RNR3*		Hughes et al. (2000) [57]
		Suppressors of telomerase insufficiency	VIII (−1)	*PRP8, UTP9, KOG1, SCH9*		Millet et al. (2016) [69]
		Tunicamycin resistance	XVI (−1), II (+1)	*ALG7, PRE7, YBR085C-A*		Chen et al. (2012) [61], Beaupere et al. (2018) [71]
		Xylose utilization	I (−1)			Sato et al. (2014) [63]
*S. paradoxus*	Environmental isolates	Freeze-thaw tolerance	XII (+1)	*AQY2*		Will et al. (2010) [84]

The fungal species, strain characteristics and selected phenotype are indicated along with the corresponding aneuploid chromosomes. In cases where specific genes on aneuploid chromosomes were implicated in the phenotype, the implicated genes are listed. If aneuploidy was confirmed to be transient, it is noted. (* —segmental aneuploidy, ** —accessory chromosome).

## References

[1] Torres E.M., Sokolsky T., Tucker C.M., Chan L.Y., Boselli M., Dunham M.J., Amon A. (2007). Effects of aneuploidy on cellular physiology and cell division in haploid yeast. Science.

[2] Williams B.R., Prabhu V.R., Hunter K.E., Glazier C.M., Whittaker C.A., Housman D.E., Amon A. (2008). Aneuploidy affects proliferation and spontaneous immortalization in mammalian cells. Science.

[3] Stingele S., Stoehr G., Peplowska K., Cox J., Mann M., Storchova Z. (2012). Global analysis of genome, transcriptome and proteome reveals the response to aneuploidy in human cells. Mol. Syst. Biol..

[4] Segal D.J., McCoy E.E. (1974). Studies on Down’s syndrome in tissue culture. I. Growth rates and protein contents of fibroblast cultures. J. Cell. Physiol..

[5] Gogendeau D., Siudeja K., Gambarotto D., Pennetier C., Bardin A.J., Basto R. (2015). Aneuploidy causes premature differentiation of neural and intestinal stem cells. Nat. Commun..

[6] Pavelka N., Rancati G., Zhu J., Bradford W.D., Saraf A., Florens L., Sanderson B.W., Hattem G.L., Li R. (2010). Aneuploidy confers quantitative proteome changes and phenotypic variation in budding yeast. Nature.

[7] Blakeslee A.F., Belling J., Farnham M.E. (1920). Chromosomal duplication and mendelian phenomena in datura mutants. Science.

[8] Satina S., Blakeslee A.F., Avery A.G. (1937). Balanced and unbalanced haploids in datura. J. Hered..

[9] Roper R.J., Reeves R.H. (2006). Understanding the basis for Down syndrome phenotypes. PLoS Genet..

[10] Beroukhim R., Mermel C.H., Porter D., Wei G., Raychaudhuri S., Donovan J., Barretina J., Boehm J.S., Dobson J., Urashima M. (2010). The landscape of somatic copy-number alteration across human cancers. Nature.

[11] Lejeune J. (1959). Etude des chromosomes somatiques de neuf enfants mongoliens. C. R. Acad. Sci..

[12] Gordon D.J., Resio B., Pellman D. (2012). Causes and consequences of aneuploidy in cancer. Nat. Rev. Genet..

[13] Hanks S., Coleman K., Reid S., Plaja A., Firth H., Fitzpatrick D., Kidd A., Méhes K., Nash R., Robin N. (2004). Constitutional aneuploidy and cancer predisposition caused by biallelic mutations in BUB1B. Nat. Genet..

[14] Duncan A.W., Hanlon Newell A.E., Smith L., Wilson E.M., Olson S.B., Thayer M.J., Strom S.C., Grompe M. (2012). Frequent aneuploidy among normal human hepatocytes. Gastroenterology.

[15] Knouse K.A., Wu J., Whittaker C.A., Amon A. (2014). Single cell sequencing reveals low levels of aneuploidy across mammalian tissues. Proc. Natl. Acad. Sci. USA.

[16] Rehen S.K., McConnell M.J., Kaushal D., Kingsbury M.A., Yang A.H., Chun J. (2001). Chromosomal variation in neurons of the developing and adult mammalian nervous system. Proc. Natl. Acad. Sci. USA.

[17] Rehen S.K., Yung Y.C., McCreight M.P., Kaushal D., Yang A.H., Almeida B.S.V., Kingsbury M.A., Cabral K.M.S., McConnell M.J., Anliker B. (2005). Constitutional aneuploidy in the normal human brain. J. Neurosci..

[18] Selmecki A., Forche A., Berman J. (2006). Aneuploidy and isochromosome formation in drug-resistant Candida albicans. Science.

[19] Hirakawa M.P., Martinez D.A., Sakthikumar S., Anderson M.Z., Berlin A., Gujja S., Zeng Q., Zisson E., Wang J.M., Greenberg J.M. (2015). Genetic and phenotypic intra-species variation in Candida albicans. Genome Res..

[20] Peter J., De Chiara M., Friedrich A., Yue J.-X., Pflieger D., Bergström A., Sigwalt A., Barre B., Freel K., Llored A. (2018). Genome evolution across 1,011 Saccharomyces cerevisiae isolates. Nature.

[21] Tan Z., Hays M., Cromie G.A., Jeffery E.W., Scott A.C., Ahyong V., Sirr A., Skupin A., Dudley A.M. (2013). Aneuploidy underlies a multicellular phenotypic switch. Proc. Natl. Acad. Sci. USA.

[22] Bennett R.J., Forche A., Berman J. (2014). Rapid mechanisms for generating genome diversity: Whole ploidy shifts, aneuploidy, and loss of heterozygosity. Cold Spring Harb. Perspect. Med..

[23] Sionov E., Lee H., Chang Y.C., Kwon-Chung K.J. (2010). Cryptococcus neoformans overcomes stress of azole drugs by formation of disomy in specific multiple chromosomes. PLoS Pathog..

[24] Berman J. (2016). Ploidy plasticity: A rapid and reversible strategy for adaptation to stress. FEMS Yeast Res..

[25] Torres E.M., Dephoure N., Panneerselvam A., Tucker C.M., Whittaker C.A., Gygi S.P., Dunham M.J., Amon A. (2010). Identification of aneuploidy-tolerating mutations. Cell.

[26] Sheltzer J.M., Blank H.M., Pfau S.J., Tange Y., George B.M., Humpton T.J., Brito I.L., Hiraoka Y., Niwa O., Amon A. (2011). Aneuploidy drives genomic instability in yeast. Science.

[27] Sheltzer J.M., Torres E.M., Dunham M.J., Amon A. (2012). Transcriptional consequences of aneuploidy. Proc. Natl. Acad. Sci. USA.

[28] Oromendia A.B., Dodgson S.E., Amon A. (2012). Aneuploidy causes proteotoxic stress in yeast. Genes Dev..

[29] Thorburn R.R., Gonzalez C., Brar G.A., Christen S., Carlile T.M., Ingolia N.T., Sauer U., Weissman J.S., Amon A. (2013). Aneuploid yeast strains exhibit defects in cell growth and passage through START. Mol. Biol. Cell.

[30] Dodgson S.E., Santaguida S., Kim S., Sheltzer J., Amon A. (2016). The pleiotropic deubiquitinase Ubp3 confers aneuploidy tolerance. Genes Dev..

[31] Tsai H.-J., Nelliat A.R., Choudhury M.I., Kucharavy A., Bradford W.D., Cook M.E., Kim J., Mair D.B., Sun S.X., Schatz M.C. (2019). Hypo-osmotic-like stress underlies general cellular defects of aneuploidy. Nature.

[32] Dephoure N., Hwang S., O’Sullivan C., Dodgson S.E., Gygi S.P., Amon A., Torres E.M. (2014). Quantitative proteomic analysis reveals posttranslational responses to aneuploidy in yeast. eLife.

[33] Blank H.M., Sheltzer J.M., Meehl C.M., Amon A. (2015). Mitotic entry in the presence of DNA damage is a widespread property of aneuploidy in yeast. Mol. Biol. Cell.

[34] Taggart J.C., Li G.-W. (2018). Production of Protein-Complex Components Is Stoichiometric and Lacks General Feedback Regulation in Eukaryotes. Cell Syst..

[35] Weinstein B., Solomon F. (1990). Phenotypic consequences of tubulin overproduction in Saccharomyces cerevisiae: Differences between alpha-tubulin and beta-tubulin. Mol. Cell. Biol..

[36] Gasch A.P., Spellman P.T., Kao C.M., Carmel-Harel O., Eisen M.B., Storz G., Botstein D., Brown P.O. (2000). Genomic expression programs in the response of yeast cells to environmental changes. Mol. Biol. Cell.

[37] Cromie G.A., Tan Z., Hays M., Jeffery E.W., Dudley A.M. (2017). Dissecting Gene Expression Changes Accompanying a Ploidy-Based Phenotypic Switch. G3.

[38] Hose J., Yong C.M., Sardi M., Wang Z., Newton M.A., Gasch A.P. (2015). Dosage compensation can buffer copy-number variation in wild yeast. eLife.

[39] Wertheimer N.B., Stone N., Berman J. (2016). Ploidy dynamics and evolvability in fungi. Philos. Trans. R. Soc. Lond. B Biol. Sci..

[40] Zhu Y.O., Siegal M.L., Hall D.W., Petrov D.A. (2014). Precise estimates of mutation rate and spectrum in yeast. Proc. Natl. Acad. Sci. USA.

[41] Ezov T.K., Boger-Nadjar E., Frenkel Z., Katsperovski I., Kemeny S., Nevo E., Korol A., Kashi Y. (2006). Molecular-genetic biodiversity in a natural population of the yeast Saccharomyces cerevisiae from “Evolution Canyon”: Microsatellite polymorphism, ploidy and controversial sexual status. Genetics.

[42] Lidzbarsky G.A., Shkolnik T., Nevo E. (2009). Adaptive response to DNA-damaging agents in natural Saccharomyces cerevisiae populations from “Evolution Canyon”, Mt. Carmel, Israel. PLoS ONE.

[43] Chang S.-L., Lai H.-Y., Tung S.-Y., Leu J.-Y. (2013). Dynamic large-scale chromosomal rearrangements fuel rapid adaptation in yeast populations. PLoS Genet..

[44] Forche A. (2014). Large-Scale Chromosomal Changes and Associated Fitness Consequences in Pathogenic Fungi. Curr. Fungal Infect. Rep..

[45] van den Bossche H., Marichal P., Odds F.C., Le Jeune L., Coene M.C. (1992). Characterization of an azole-resistant Candida glabrata isolate. Antimicrob. Agents Chemother..

[46] Marichal P., Vanden Bossche H., Odds F.C., Nobels G., Warnock D.W., Timmerman V., Van Broeckhoven C., Fay S., Mose-Larsen P. (1997). Molecular biological characterization of an azole-resistant Candida glabrata isolate. Antimicrob. Agents Chemother..

[47] Poláková S., Blume C., Zárate J.A., Mentel M., Jørck-Ramberg D., Stenderup J., Piskur J. (2009). Formation of new chromosomes as a virulence mechanism in yeast Candida glabrata. Proc. Natl. Acad. Sci. USA.

[48] Selmecki A., Gerami-Nejad M., Paulson C., Forche A., Berman J. (2008). An isochromosome confers drug resistance in vivo by amplification of two genes, ERG11 and TAC1. Mol. Microbiol..

[49] Ford C.B., Funt J.M., Abbey D., Issi L., Guiducci C., Martinez D.A., Delorey T., Li B.Y., White T.C., Cuomo C. (2015). The evolution of drug resistance in clinical isolates of Candida albicans. eLife.

[50] Selmecki A.M., Dulmage K., Cowen L.E., Anderson J.B., Berman J. (2009). Acquisition of aneuploidy provides increased fitness during the evolution of antifungal drug resistance. PLoS Genet..

[51] Bravo Ruiz G., Ross Z.K., Holmes E., Schelenz S., Gow N.A.R., Lorenz A. (2019). Rapid and extensive karyotype diversification in haploid clinical Candida auris isolates. Curr. Genet..

[52] Ngamskulrungroj P., Chang Y., Hansen B., Bugge C., Fischer E., Kwon-Chung K.J. (2012). Characterization of the chromosome 4 genes that affect fluconazole-induced disomy formation in Cryptococcus neoformans. PLoS ONE.

[53] Sionov E., Chang Y.C., Kwon-Chung K.J. (2013). Azole heteroresistance in Cryptococcus neoformans: Emergence of resistant clones with chromosomal disomy in the mouse brain during fluconazole treatment. Antimicrob. Agents Chemother..

[54] Gerstein A.C., Fu M.S., Mukaremera L., Li Z., Ormerod K.L., Fraser J.A., Berman J., Nielsen K. (2015). Polyploid titan cells produce haploid and aneuploid progeny to promote stress adaptation. mBio.

[55] Zhu Y.O., Sherlock G., Petrov D.A. (2016). Whole Genome Analysis of 132 Clinical Saccharomyces cerevisiae Strains Reveals Extensive Ploidy Variation. G3.

[56] Forche A., Solis N.V., Swidergall M., Thomas R., Guyer A., Beach A., Cromie G.A., Le G.T., Lowell E., Pavelka N. (2019). Selection of Candida albicans trisomy during oropharyngeal infection results in a commensal-like phenotype. PLoS Genet..

[57] Hughes T.R., Roberts C.J., Dai H., Jones A.R., Meyer M.R., Slade D., Burchard J., Dow S., Ward T.R., Kidd M.J. (2000). Widespread aneuploidy revealed by DNA microarray expression profiling. Nat. Genet..

[58] Gasch A.P., Huang M., Metzner S., Botstein D., Elledge S.J., Brown P.O. (2001). Genomic expression responses to DNA-damaging agents and the regulatory role of the yeast ATR homolog Mec1p. Mol. Biol. Cell.

[59] Gresham D., Desai M.M., Tucker C.M., Jenq H.T., Pai D.A., Ward A., DeSevo C.G., Botstein D., Dunham M.J. (2008). The repertoire and dynamics of evolutionary adaptations to controlled nutrient-limited environments in yeast. PLoS Genet..

[60] Rancati G., Pavelka N., Fleharty B., Noll A., Trimble R., Walton K., Perera A., Staehling-Hampton K., Seidel C.W., Li R. (2008). Aneuploidy underlies rapid adaptive evolution of yeast cells deprived of a conserved cytokinesis motor. Cell.

[61] Chen G., Bradford W.D., Seidel C.W., Li R. (2012). Hsp90 stress potentiates rapid cellular adaptation through induction of aneuploidy. Nature.

[62] Yona A.H., Manor Y.S., Herbst R.H., Romano G.H., Mitchell A., Kupiec M., Pilpel Y., Dahan O. (2012). Chromosomal duplication is a transient evolutionary solution to stress. Proc. Natl. Acad. Sci. USA.

[63] Sato T.K., Liu T., Parreiras L.S., Williams D.L., Wohlbach D.J., Bice B.D., Ong I.M., Breuer R.J., Qin L., Busalacchi D. (2014). Harnessing genetic diversity in Saccharomyces cerevisiae for fermentation of xylose in hydrolysates of alkaline hydrogen peroxide-pretreated biomass. Appl. Environ. Microbiol..

[64] Sunshine A.B., Payen C., Ong G.T., Liachko I., Tan K.M., Dunham M.J. (2015). The fitness consequences of aneuploidy are driven by condition-dependent gene effects. PLoS Biol..

[65] Gerstein A.C., Ono J., Lo D.S., Campbell M.L., Kuzmin A., Otto S.P. (2015). Too much of a good thing: The unique and repeated paths toward copper adaptation. Genetics.

[66] Selmecki A.M., Maruvka Y.E., Richmond P.A., Guillet M., Shoresh N., Sorenson A.L., De S., Kishony R., Michor F., Dowell R. (2015). Polyploidy can drive rapid adaptation in yeast. Nature.

[67] Sirr A., Cromie G.A., Jeffery E.W., Gilbert T.L., Ludlow C.L., Scott A.C., Dudley A.M. (2015). Allelic variation, aneuploidy, and nongenetic mechanisms suppress a monogenic trait in yeast. Genetics.

[68] Voordeckers K., Kominek J., Das A., Espinosa-Cantú A., De Maeyer D., Arslan A., Van Pee M., van der Zande E., Meert W., Yang Y. (2015). Adaptation to High Ethanol Reveals Complex Evolutionary Pathways. PLoS Genet..

[69] Millet C., Makovets S. (2016). Aneuploidy as a mechanism of adaptation to telomerase insufficiency. Curr. Genet..

[70] Hope E.A., Amorosi C.J., Miller A.W., Dang K., Heil C.S., Dunham M.J. (2017). Experimental Evolution Reveals Favored Adaptive Routes to Cell Aggregation in Yeast. Genetics.

[71] Beaupere C., Dinatto L., Wasko B.M., Chen R.B., VanValkenburg L., Kiflezghi M.G., Lee M.B., Promislow D.E.L., Dang W., Kaeberlein M. (2018). Genetic screen identifies adaptive aneuploidy as a key mediator of ER stress resistance in yeast. Proc. Natl. Acad. Sci. USA.

[72] Lauer S., Avecilla G., Spealman P., Sethia G., Brandt N., Levy S.F., Gresham D. (2018). Single-cell copy number variant detection reveals the dynamics and diversity of adaptation. PLoS Biol..

[73] Dunham M.J., Badrane H., Ferea T., Adams J., Brown P.O., Rosenzweig F., Botstein D. (2002). Characteristic genome rearrangements in experimental evolution of Saccharomyces cerevisiae. Proc. Natl. Acad. Sci. USA.

[74] Pavelka N., Rancati G., Li R. (2010). Dr Jekyll and Mr Hyde: Role of aneuploidy in cellular adaptation and cancer. Curr. Opin. Cell Biol..

[75] Yang F., Kravets A., Bethlendy G., Welle S., Rustchenko E. (2013). Chromosome 5 monosomy of Candida albicans controls susceptibility to various toxic agents, including major antifungals. Antimicrob. Agents Chemother..

[76] Yang F., Teoh F., Tan A.S.M., Cao Y., Pavelka N., Berman J. (2019). Aneuploidy Enables Cross-Adaptation to Unrelated Drugs. Mol. Biol. Evol..

[77] Mount H.O., Revie N.M., Todd R.T., Anstett K., Collins C., Costanzo M., Boone C., Robbins N., Selmecki A., Cowen L.E. (2018). Global analysis of genetic circuitry and adaptive mechanisms enabling resistance to the azole antifungal drugs. PLoS Genet..

[78] Ma L.-J., van der Does H.C., Borkovich K.A., Coleman J.J., Daboussi M.-J., Di Pietro A., Dufresne M., Freitag M., Grabherr M., Henrissat B. (2010). Comparative genomics reveals mobile pathogenicity chromosomes in Fusarium. Nature.

[79] Miao V.P., Covert S.F., VanEtten H.D. (1991). A fungal gene for antibiotic resistance on a dispensable (“B”) chromosome. Science.

[80] van den Broek M., Bolat I., Nijkamp J.F., Ramos E., Luttik M.A.H., Koopman F., Geertman J.M., de Ridder D., Pronk J.T., Daran J.-M. (2015). Chromosomal Copy Number Variation in Saccharomyces pastorianus Is Evidence for Extensive Genome Dynamics in Industrial Lager Brewing Strains. Appl. Environ. Microbiol..

[81] Morard M., Macías L.G., Adam A.C., Lairón-Peris M., Pérez-Torrado R., Toft C., Barrio E. (2019). Aneuploidy and Ethanol Tolerance in Saccharomyces cerevisiae. Front. Genet..

[82] Guijo S., Mauricio J.C., Salmon J.M., Ortega J.M. (1997). Determination of the relative ploidy in different Saccharomyces cerevisiae strains used for fermentation and “flor”film ageing of dry sherry-type wines. Yeast.

[83] Zhang K., Zhang L.-J., Fang Y.-H., Jin X.-N., Qi L., Wu X.-C., Zheng D.-Q. (2016). Genomic structural variation contributes to phenotypic change of industrial bioethanol yeast Saccharomyces cerevisiae. FEMS Yeast Res..

[84] Will J.L., Kim H.S., Clarke J., Painter J.C., Fay J.C., Gasch A.P. (2010). Incipient balancing selection through adaptive loss of aquaporins in natural Saccharomyces cerevisiae populations. PLoS Genet..

[85] Coste A., Turner V., Ischer F., Morschhäuser J., Forche A., Selmecki A., Berman J., Bille J., Sanglard D. (2006). A mutation in Tac1p, a transcription factor regulating CDR1 and CDR2, is coupled with loss of heterozygosity at chromosome 5 to mediate antifungal resistance in Candida albicans. Genetics.

[86] Coste A., Selmecki A., Forche A., Diogo D., Bougnoux M.-E., d’Enfert C., Berman J., Sanglard D. (2007). Genotypic evolution of azole resistance mechanisms in sequential Candida albicans isolates. Eukaryot. Cell.

[87] Perea S., López-Ribot J.L., Kirkpatrick W.R., McAtee R.K., Santillán R.A., Martínez M., Calabrese D., Sanglard D., Patterson T.F. (2001). Prevalence of molecular mechanisms of resistance to azole antifungal agents in Candida albicans strains displaying high-level fluconazole resistance isolated from human immunodeficiency virus-infected patients. Antimicrob. Agents Chemother..

[88] Liu T.T., Znaidi S., Barker K.S., Xu L., Homayouni R., Saidane S., Morschhäuser J., Nantel A., Raymond M., Rogers P.D. (2007). Genome-wide expression and location analyses of the Candida albicans Tac1p regulon. Eukaryot. Cell.

[89] Harrison B.D., Hashemi J., Bibi M., Pulver R., Bavli D., Nahmias Y., Wellington M., Sapiro G., Berman J. (2014). A tetraploid intermediate precedes aneuploid formation in yeasts exposed to fluconazole. PLoS Biol..

[90] Muller H., Thierry A., Coppée J.-Y., Gouyette C., Hennequin C., Sismeiro O., Talla E., Dujon B., Fairhead C. (2009). Genomic polymorphism in the population of Candida glabrata: Gene copy-number variation and chromosomal translocations. Fungal Genet. Biol..

[91] Chang Y.C., Khanal Lamichhane A., Kwon-Chung K.J. (2018). Cryptococcus neoformans, Unlike Candida albicans, Forms Aneuploid Clones Directly from Uninucleated Cells under Fluconazole Stress. mBio.

[92] Altamirano S., Fang D., Simmons C., Sridhar S., Wu P., Sanyal K., Kozubowski L. (2017). Fluconazole-Induced Ploidy Change in Cryptococcus neoformans Results from the Uncoupling of Cell Growth and Nuclear Division. mSphere.

[93] Bennett R.J., Johnson A.D. (2003). Completion of a parasexual cycle in Candida albicans by induced chromosome loss in tetraploid strains. EMBO J..

[94] Forche A., Alby K., Schaefer D., Johnson A.D., Berman J., Bennett R.J. (2008). The parasexual cycle in Candida albicans provides an alternative pathway to meiosis for the formation of recombinant strains. PLoS Biol..

[95] Berman J., Hadany L. (2012). Does stress induce (para) sex? Implications for Candida albicans evolution. Trends Genet..

[96] Hickman M.A., Paulson C., Dudley A., Berman J. (2015). Parasexual Ploidy Reduction Drives Population Heterogeneity through Random and Transient Aneuploidy in Candida albicans. Genetics.

[97] Todd R.T., Wikoff T.D., Forche A., Selmecki A. (2019). Genome plasticity in Candida albicans is driven by long repeat sequences. eLife.

[98] Payen C., Di Rienzi S.C., Ong G.T., Pogachar J.L., Sanchez J.C., Sunshine A.B., Raghuraman M.K., Brewer B.J., Dunham M.J. (2014). The dynamics of diverse segmental amplifications in populations of Saccharomyces cerevisiae adapting to strong selection. G3.

[99] Möller M., Stukenbrock E.H. (2017). Evolution and genome architecture in fungal plant pathogens. Nat. Rev. Microbiol..

[100] Hu J., Shrestha S., Zhou Y., Liu X., Lamour K. (2018). Dynamic Extreme Aneuploidy (DEA) in the vegetable pathogen Phytophthora capsici sheds light on instant evolution and intractability. BioRxiv.

[101] Kasuga T., Bui M., Bernhardt E., Swiecki T., Aram K., Cano L.M., Webber J., Brasier C., Press C., Grünwald N.J. (2016). Host-induced aneuploidy and phenotypic diversification in the Sudden Oak Death pathogen Phytophthora ramorum. BMC Genom..

[102] Mulla W.A., Seidel C.W., Zhu J., Tsai H.-J., Smith S.E., Singh P., Bradford W.D., McCroskey S., Nelliat A.R., Conkright J. (2017). Aneuploidy as a cause of impaired chromatin silencing and mating-type specification in budding yeast. eLife.

[103] Liu G., Yong M.Y.J., Yurieva M., Srinivasan K.G., Liu J., Lim J.S.Y., Poidinger M., Wright G.D., Zolezzi F., Choi H. (2015). Gene Essentiality Is a Quantitative Property Linked to Cellular Evolvability. Cell.

[104] Beach R.R., Ricci-Tam C., Brennan C.M., Moomau C.A., Hsu P.-H., Hua B., Silberman R.E., Springer M., Amon A. (2017). Aneuploidy Causes Non-genetic Individuality. Cell.

[105] Stirling P.C., Bloom M.S., Solanki-Patil T., Smith S., Sipahimalani P., Li Z., Kofoed M., Ben-Aroya S., Myung K., Hieter P. (2011). The complete spectrum of yeast chromosome instability genes identifies candidate CIN cancer genes and functional roles for ASTRA complex components. PLoS Genet..

[106] Zhu J., Pavelka N., Bradford W.D., Rancati G., Li R. (2012). Karyotypic determinants of chromosome instability in aneuploid budding yeast. PLoS Genet..

[107] Duffy S., Fam H.K., Wang Y.K., Styles E.B., Kim J.-H., Ang J.S., Singh T., Larionov V., Shah S.P., Andrews B. (2016). Overexpression screens identify conserved dosage chromosome instability genes in yeast and human cancer. Proc. Natl. Acad. Sci. USA.

[108] Tutaj H., Pogoda E., Tomala K., Korona R. (2019). Gene overexpression screen for chromosome instability in yeast primarily identifies cell cycle progression genes. Curr. Genet..

[109] Stirling P.C., Crisp M.J., Basrai M.A., Tucker C.M., Dunham M.J., Spencer F.A., Hieter P. (2012). Mutability and mutational spectrum of chromosome transmission fidelity genes. Chromosoma.

[110] Zhu J., Heinecke D., Mulla W.A., Bradford W.D., Rubinstein B., Box A., Haug J.S., Li R. (2015). Single-Cell Based Quantitative Assay of Chromosome Transmission Fidelity. G3.

[111] Ravichandran M.C., Fink S., Clarke M.N., Hofer F.C., Campbell C.S. (2018). Genetic interactions between specific chromosome copy number alterations dictate complex aneuploidy patterns. Genes Dev..

[112] Tange Y., Kurabayashi A., Goto B., Hoe K.-L., Kim D.-U., Park H.-O., Hayles J., Chikashige Y., Tsutumi C., Hiraoka Y. (2012). The CCR4-NOT complex is implicated in the viability of aneuploid yeasts. PLoS Genet..

[113] Dodgson S.E., Kim S., Costanzo M., Baryshnikova A., Morse D.L., Kaiser C.A., Boone C., Amon A. (2016). Chromosome-Specific and Global Effects of Aneuploidy in Saccharomyces cerevisiae. Genetics.

[114] Chen G., Mulla W.A., Kucharavy A., Tsai H.-J., Rubinstein B., Conkright J., McCroskey S., Bradford W.D., Weems L., Haug J.S. (2015). Targeting the adaptability of heterogeneous aneuploids. Cell.

